# Energy‐Based Devices for the Treatment of Cutaneous Lesions in Patients With Lupus Erythematosus and Dermatomyositis

**DOI:** 10.1111/jocd.70642

**Published:** 2026-01-12

**Authors:** Hae Chang Joh, Mihn‐Sook Jue, Joo Yeon Ko

**Affiliations:** ^1^ Department of Dermatology College of Medicine, University of Hanyang Seoul Korea

**Keywords:** dermatomyositis, energy‐based devices, lupus erythematosus

## Abstract

**Background:**

The use of energy‐based devices (EBDs) for aesthetic and dermatological conditions is increasing, but data on efficacy and safety in autoimmune connective tissue disease (CTD) patients remain limited.

**Objectives:**

This study assesses EBD real‐world treatment outcomes in Korean patients with lupus erythematosus (LE) and dermatomyositis (DM).

**Methods:**

A retrospective, uncontrolled study was conducted on 26 CTD patients (LE: 20, DM: 6) treated at Hanyang University Seoul Hospital (2015–2023). Various laser modalities, including pulsed‐dye laser (PDL), intense pulsed light (IPL), long‐pulse Nd:YAG (LPNY), Q‐switched Nd:YAG (QSNY), and ablative fractional laser (AFL), were analyzed. Two independent dermatologists evaluated treatment outcomes using a 5‐point modified Investigator's Global Assessment (IGA) scale.

**Results:**

Patients showed significant improvement in erythema, dyspigmentation, and overall skin morphology. Some LE patients also exhibited enhanced follicular activity. Treatments were well‐tolerated, with only transient side effects reported, and no long‐term complications or disease reactivation occurred.

**Conclusions:**

EBD treatments may provide cosmetic improvement in selected LE and DM patients and are generally safe. Larger controlled studies are needed to confirm efficacy and establish optimal protocols.

## 1 Introduction

Energy‐based devices (EBDs) are increasingly used for various aesthetic and dermatologic applications. Although generally considered safe with mild and transient side effects, laser treatments are perceived as risky in patients with underlying immunologic deficits or autoimmune connective tissue diseases (CTDs) owing to potential impairments in the wound healing process and an increased risk of laser site infection [1].

The first reported use of laser treatment for CTD was CO_2_ laser to discoid lupus erythematosus (DLE) lesions in 1986 [2]. Since then, various treatments, including argon lasers, intense pulsed light (IPL), Erbium‐doped yttrium aluminum garnet (Er:YAG) lasers, neodymium‐doped yttrium aluminum garnet (Nd:YAG) lasers, and pulsed‐dye lasers (PDLs) have been used to treat lupus erythematosus (LE) lesions [3, 4, 5].

Most of the studies on laser treatment in patients with CTD are related to cutaneous LE (CLE). However, some studies have also highlighted the successful treatment of erythema, telangiectasia, poikiloderma, and induration in patients with dermatomyositis (DM) using laser therapy, without any significant adverse effects. Nevertheless, most of these studies are confined to case studies or series with small sample sizes, particularly in the case of DM. Moreover, no study has examined the use of various laser combinations in CTD treatment [6, 7, 8]

CTD, particularly CLE, can significantly impact a patient's physical appearance and is often refractory to many first‐ and second‐line therapies. The cosmetic implications of CTD, especially when it manifests in visible areas such as the face and upper extremities, can lead to a profound impairment of a patient's quality of life (QoL) and psychological well‐being [9, 10].

In this retrospective study, we aimed to evaluate the outcomes in Korean patients with CTD, specifically LE and DM, who underwent cosmetic treatment using EBDs.

## 2 Materials and Methods

### 2.1 Study Design and Patients

In this retrospective study, we conducted an analysis of patients with CTD who visited the Hanyang University Seoul Hospital in Korea. Given Hanyang University's specialization in rheumatology, a substantial number of CTD patients regularly seek care at the dermatology department.

**FIGURE 1:**
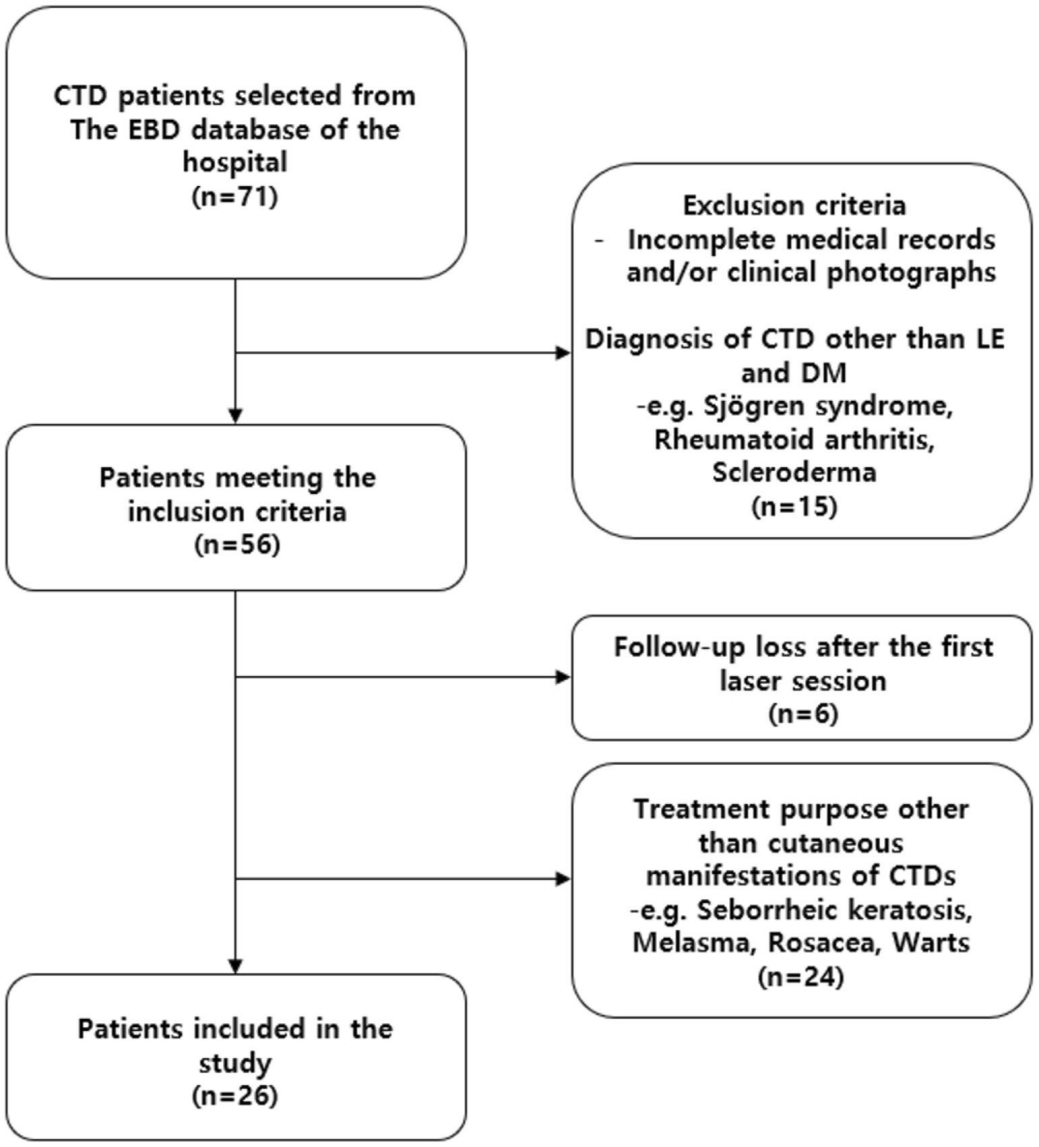
Flowchart of the patient enrollment.

All EBD treatments in our department are documented in our EBD database, covering the period from 2015 to the present date. From the pool of CTD patients identified within the database, only those meeting specific inclusion criteria were considered for analysis. These criteria included: (1) Patients who were clinically and/or histologically diagnosed with CLE, SLE, and DM. The diagnosis of concurrent SLE was established when patients met the ACR/EULAR 2019 Classification criteria at the time of their visit to the dermatologist [11]. (2) Patients who underwent laser treatment at least once during December 2015–November 2023. (3) Patients with clinical photographs suitable for outcome evaluation. Patients were excluded if their medical records or clinical photographs lacked essential information for the variables of interest (Figure 1).

This study was reviewed and approved by the Institutional Review Board (IRB) of Hanyang University Seoul Hospital (IRB no. HYUH 2023–11‐045).

### 2.2 Methods

We retrospectively assessed the patients' medical records and demographic information, including sex, age, diagnosis, concurrence of SLE, location of the cutaneous lesion, previous and concomitant treatments, and the occurrence of adverse effects during the follow‐up period in months. The follow‐up period in months was defined as the time from the first laser treatment session to the date of the final follow‐up.

#### 2.2.1 Energy‐Based Devices

Detailed laser modalities, parameters, treatment intervals, and number of treatment sessions are provided in Table S4.

#### 2.2.2 Clinical Assessment

The clinical efficacy of the treatments was evaluated using a novel method inspired by the Cutaneous Lupus Activity Investigator's Global Assessment (CLA‐IGA) scoring system [12], which was modified to better align with the variables of interest in this study. Our modified IGA consisted of three scales: erythema, dyspigmentation, and other morphological characteristics (OMCs; global impression with regard to scale, edema/infiltration, and/or secondary change [vesicle, erosion, crusting, scarring, atrophy]), each ranging from 0–4.

The degree of erythema (0 = none, 1 = faint, 2 = pink/mild, 3 = red, 4 = violaceous/bright red) was evaluated in patients treated with vascular lasers (PDL and LPNY laser), and in some cases, IPL and AFLs were used to address erythema components. Dyspigmentation (0 = none, 1 = faint, 2 = light brown/mild, 3 = brown, 4 = dark brown) was evaluated in patients who were treated with pigment lasers (QSNY laser). In some cases, IPL and AFLs were used to address pigmentation. OMC (0 = none, 1 = minimal, 2 = mild, 3 = moderate, 4 = severe) were evaluated in all patients. In cases of LE, an additional scale was employed to assess improvement in follicular activity, with a score of 0 indicating no improvement and 1 indicating improvement. Follicular activity was defined as the presence of follicular plugging or hyperkeratosis in the lesion. This modified IGA was adopted to allow standardized retrospective assessment across heterogeneous clinical presentations. Although it enabled consistent evaluation, the lack of external validation may limit comparability with other studies.

Although each laser (PDL, LPNY laser, IPL, QSNY laser, and AFLs) has multifaceted effects (vascular changes, pigment alterations, and skin texture improvements), for clarity, we categorized and analyzed the efficacy of lasers based on its primary effects.

All cases were photographed, and two independent dermatologists reviewed the pretreatment (photographs before the first session of laser treatment) and posttreatment photographs (photographs after the last session of laser therapy) to assess the treatment response. Inter‐rater agreement was assessed qualitatively and showed acceptable concordance.

#### 2.2.3 Patient Satisfaction and Adverse Events

Through a retrospective chart review, patient satisfaction was evaluated using a visual analog scale (VAS), where 0 represented a fully unsatisfactory result, and 10 represented an excellent posttreatment cosmetic outcome. Any adverse events related to the laser treatment, including dyspigmentation, scarring, purpura, prolonged pain/swelling, prolonged erythema, blisters, or exacerbation of cutaneous or systemic diseases, were documented during the follow‐up period.

#### 2.2.4 Statistical Analysis

All statistical analyses were performed using SPSS Statistics 25.0 (IBM Co., Armonk, NY, USA). Absolute and relative frequencies were calculated for the qualitative variables. To compare the differences between the pre and posttreatment IGA scores, the Wilcoxon signed‐rank test was performed. Statistical significance was set at *p* < 0.05. Given the small sample size, particularly in DM patients, effect sizes and confidence intervals were additionally calculated where applicable.

## 3 Results

### 3.1 Patient Characteristics

Twenty‐six patients with CTD were included in this study. Among them, 18 were female (69.2%), and the mean age at enrollment was 33.77 ± 9.98 years. Twenty patients were diagnosed with LE and six with DM. When classified by Fitzpatrick skin type, nine were skin type III (34.6%), and 17 were skin type IV (65.4%). Detailed patient characteristics are provided in Table 1.

**TABLE 1:** Demographics and characteristics of patients with CTD.

	CTD patients, total (*n* = 26) *n* (%)	LE patients (*n* = 20) *n* (%)	DM patients (*n* = 6) *n* (%)
Age, mean ± SD	33.77 ± 9.98	34.35 ± 7.56	31.83 ± 16.58
Sex			
Male	8 (30.8%)	6 (30.0%)	2 (33.3%)
Female	18 (69.2%)	14 (70.0%)	4 (66.7%)
Fitzpatrick skin type			
III	9 (34.6%)	8 (40.0%)	1 (16.7%)
IV	17 (65.4%)	12 (60.0%)	5 (83.3%)
Number of sessions, mean ± SD (minimum 2, maximum 18)	6.88 ± 4.48	7.40 ± 4.83	5.16 ± 2.63
Intervals between sessions (weeks), median (IQR, Q1–Q3)	8 (5–13)	8 (5–12)	9 (6–22)
Type of treatment			
PDL	21 (80.8%)	17 (85.0%)	4 (66.7%)
IPL	4 (15.4%)	4 (20.0%)	0 (0.0%)
LPNY laser	8 (30.8%)	6 (30.0%)	1 (16.7%)
QSNY laser	7 (26.9%)	5 (25.0%)	2 (33.3%)
CO_2_ AFL	6 (23.1%)	5 (25.0%)	1 (16.7%)
Er:YAG AFL	2 (7.7%)	2 (10.0%)	0 (0.0%)
Follow‐up time months, median (IQR, Q1–Q3)	17 (9–35)	17 (9–32)	29 (10–64)

### 3.2 Clinical Characteristics of Patients With LE

The clinical characteristics of patients with LE are summarized in Table 2.

**TABLE 2:** Characteristics of patients with LE.

Patient	Age	Sex	Diagnosis	Previous treatment	Concomitant treatment	EBD modalities and number of treatment	Side effect	Follow‐up duration (months)
1	31	M	CCLE	HCQ, PDS, CSA, MTX	HCQ TCI TA, PDRN ILI	IPL 1, LPNY 2, PDL 9	—	38
2	41	F	CCLE	HCQ TCI TA & H‐lase ILI	HCQ TCI	IPL 1, CO_2_ AFL 8, Er:YAG AFL 2, 532 nm QSNY 2	—	95
3	36	F	CCLE	HCQ TCI	HCQ TCI	LPNY 10, IPL 7, PDL 6	Bullae (LPNY)	47
4	34	F	CCLE, SLE	HCQ, MTX, AZA, BEL, TAC	HCQ TCS, TCI	IPL 3, LPNY 2, 532 nm QSNY 2, PDL 2	Purpura (PDL)	35
5	27	M	CCLE	HCQ TCI TA ILI	HCQ TCI PDRN ILI	LPNY 3, PDL 4	—	22
6	54	M	CCLE	HCQ TCS, TCI TA ILI	HCQ TCS, TCI TA ILI	LPNY 1, PDL 7, CO_2_ AFL 6	—	27
7	23	F	CCLE	HCQ, CSA, PDS, AZA TCI HA, TA, PDRN ILI	HCQ, AZA, PDS TCI TA, PDRN ILI	532 nm QSNY 1, Er:YAG AFL 1, PDL 16	Transient hyperpigmentation (Er:YAG AFL)	29
8	37	F	SLE telangiectasia	HCQ, AZA, TAC TCI	HCQ, TAC TCI	LPNY 9, PDL 5	—	33
9	22	F	CCLE, SLE	HCQ, MTX, TCS, TCI TA ILI	HCQ, PDS TA ILI	CO_2_ AFL 5, PDL 5, 532 nm QSNY 4	—	21
10	27	F	CCLE, SLE	HCQ TCS, TCI TA ILI	HCQ, PDS	CO_2_ AFL 2, PDL 9	—	18
11	40	M	CCLE, SLE	MMF, PDS TCI	MMF, PDS TCI TA ILI	CO_2_ AFL 4	—	9
12	32	M	CCLE, SLE	HCQ, PDS, TAC, MMF, CSA TCI, TCS H‐lase ILI	HCQ, MMF, PDS TCS TA, PDRN ILI	PDL 3	—	16
13	30	F	SLE telangiectasia	HCQ, PDS, CSA, MMF TCI, TCS	HCQ, PDS, MMF TCI	PDL 4	—	13
14	29	F	CCLE, SLE	HCQ TCI	HCQ	PDL 9	—	13
15	40	M	CCLE	HCQ TCI, TCS TA ILI	HCQ TCI TA ILI	PDL 2	—	12
16	36	F	CCLE, SLE	HCQ, AZA, TAC, CSA TCI TA ILI	HCQ, CSA, PDS TCI	PDL 2	—	8
17	45	F	SLE telangiectasia	AZA, HCQ, PDS, MTX HQ, TCI, TCS	HCQ, MMF TCS, TCI	PDL 2	—	6
18	37	F	CCLE, SLE	HCQ, PDS TCI	HCQ, PDS TCS	532 nm QSNY 6	—	7
19	34	F	SLE telangiectasia	HCQ, PDS, MTX, TAC TCI	HCQ, PDS, MTX, TAC TCI	PDL 4	—	9
20	32	F	CCLE, SLE	HCQ, PDS, MMF TCS, TCI TA ILI	HCQ, PDS, MMF TCS, TCI TA ILI	PDL 3	—	3

Of the 20 patients with LE, 13 (65.0%) were diagnosed with SLE. In this group, nine patients had concurrent chronic CLE (CCLE) and SLE, and four were treated for persistent telangiectasia Table S1. All patients applied topical corticosteroids or calcineurin inhibitors for several years, and 18 of 20 patients (90.0%) were taking hydroxychloroquine as a concomitant oral medication.

### 3.3 Clinical Outcomes of Patients With LE

**FIGURE 2:**
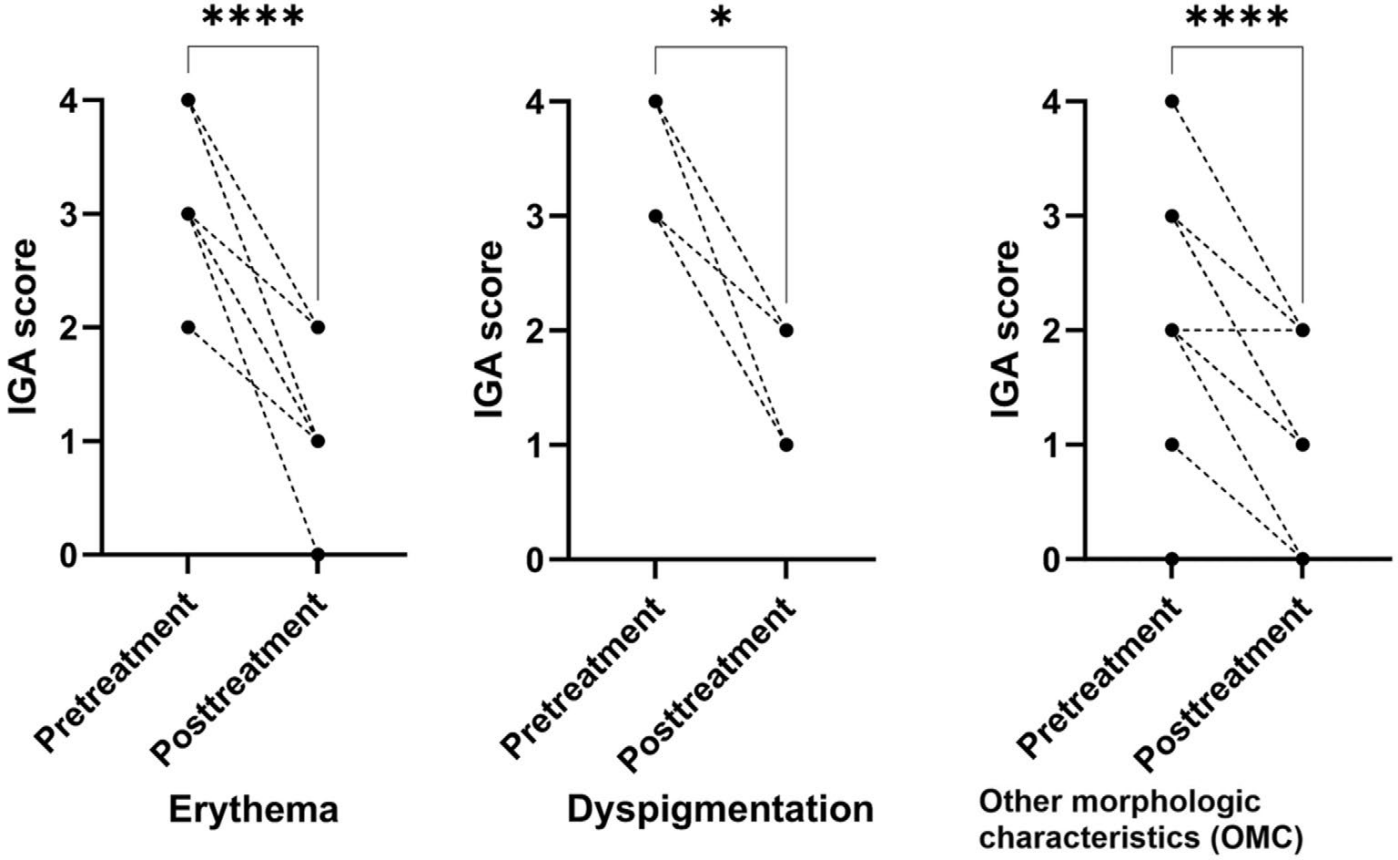
Clinical outcomes of patients with lupus erythematosus (LE) (*n* = 20). Changes of Investigator's Global Assessment (IGA) scores (erythema, dyspigmentation, other morphological characteristics) before and after the treatment sessions. *Statistically significant (*p* < 0.05) compared with baseline.

The clinical outcomes of patients with LE are presented in Figure 2. Separate IGA scores for erythema, dyspigmentation, and OMCs were evaluated. Eighteen patients were evaluated for erythema, and most patients exhibited significant improvement in the treated lesions. The median (IQR) IGA score for erythema decreased significantly from pretreatment (3 [Q1–Q3: 3–4]) to posttreatment (1 [Q1–Q3: 1–1]) (*p* < 0.001). Seven patients were evaluated for dyspigmentation, and we also observed a significant improvement in the median (IQR) IGA score from pretreatment (3 [Q1–Q3: 3–4]) to posttreatment (1 [Q1–Q3: 1–2]) (*p* = 0.014). When assessing the OMCs in all patients with LE, the median (IQR) IGA score decreased significantly from 2.5 (Q1–Q3: 2–3) at pretreatment to 1 (Q1–Q3: 0.25–1) at posttreatment (*p* < 0.001). Furthermore, we observed improvements in follicular activity in eight patients (40.0%).

### 3.4 Adverse Events and Cosmetic Result of Patients With LE

**FIGURE 3:**
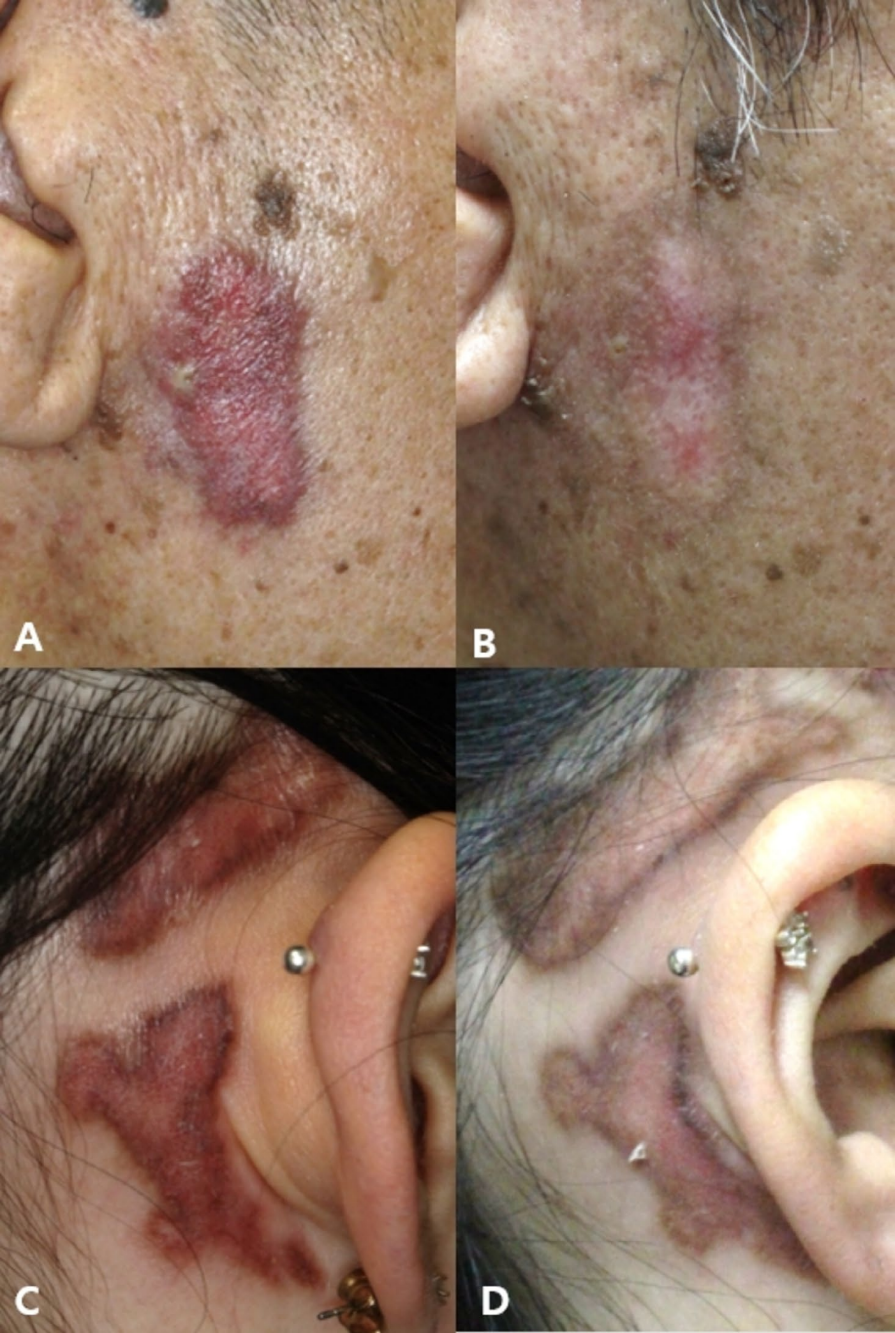
Clinical photographs of DLE lesions of patient 6 (A, B) and 9 (C, D). (A, C) At baseline, DLE lesions of two patients showed erythematous plaques, dyspigmentation with textural changes. (B, D) Marked improvement in erythema, pigmentation with OMCs, and follicular activity following combination EBD treatments at follow‐up (B: 24 months, D: 18 months).

**FIGURE 4:**
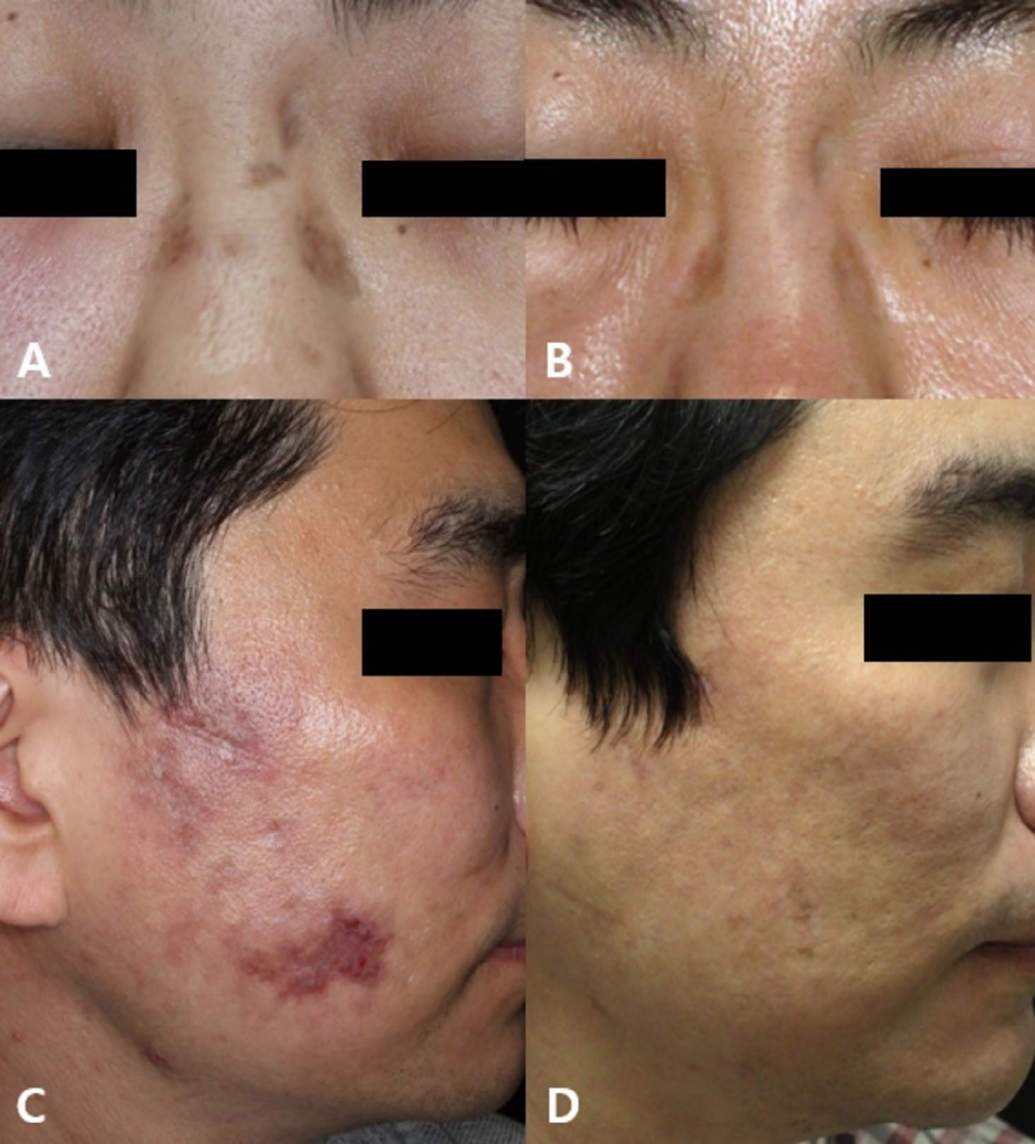
Clinical photographs of DLE lesions of patient 2 (A, B) and patient 11 (C, D). (A, C) At baseline, DLE lesions showed dyspigmentation, atrophy, and textural changes. (B, D) Marked improvement in pigmentation and lesion morphology following combination EBD treatments at follow‐up (B: 6 years, D: 9 months).

Overall, the treatments were well‐tolerated, yielding favorable results for erythema, dyspigmentation, OMCs, and follicular activity in several patients (Figures 3 and 4).

Few side effects were reported after laser treatment. One patient (skin type IV) developed transient hyperpigmentation after treatment with Er:YAG AFL. Another patient experienced bullae formation after treatment with LPNY laser. Additionally, one patient developed purpura following PDL treatment. However, these adverse effects were transient, did not lead to further complications, and did not affect the overall treatment process.

The median (IQR) follow‐up period for patients with LE was 17 (Q1–Q3: 9–32) months. During the follow‐up period, none of the patients exhibited any long‐lasting adverse effects or disease reactivation.

In terms of patient‐reported cosmetic outcomes, VAS data were available for 10 patients with LE. The overall cosmetic outcome, as measured using the VAS, was 6.66 ± 2.0, indicating a fair level of satisfaction with the cosmetic outcome of the laser treatments.

### 3.5 Clinical Characteristics of Patients With DM

The clinical characteristics of patients with DM are shown in Table 3. Persistent facial erythema and postinflammatory hyperpigmentation were treated in patients with DM (Supplementary Table 2).

**TABLE 3:** Characteristics of patients with DM.

Patient	Age	Sex	Diagnosis	Previous treatment	Concomitant treatment	EBD modalities and number of treatment	Side effect	Follow‐up duration (months)
1	49	F	Adult DM	MTX, HCQ, PDS TCI	MTX, HCQ, PDS TCI	1064 nm QSNY 3	—	45
2	24	M	Juvenile DM	MTX, HCQ, PDS, CSA TCI, TCS	MTX, HCQ, PDS, CSA TCI	CO_2_ AFL 2, PDL 4, LPNY 2	—	60
3	17	M	Juvenile DM	MTX, HCQ, PDS, CLC TCI	MTX, HCQ TCI	LPNY 1, PDL 1	—	78
4	11	F	Juvenile DM	MTX, HCQ, PDS TCI, TCS TA ILI	MTX, HCQ, PDS TCI TA ILI	PDL 7	—	14
5	42	F	Adult DM	HCQ, PDS, CSA, CLC TCI, TCS TA ILI	HCQ, PDS, CSA TCI TA ILI	PDL 3	—	12
6	48	F	Adult DM	MTX, PDS, CSA HQ, TCI, TCS	MTX, PDS, CSA HQ, TCI	1064 nm QSNY 5	—	5

All patients applied topical calcineurin inhibitors for several years and were taking at least one immunosuppressive medication. None of the patients had active muscle disease at the time of laser treatment.

### 3.6 Clinical Outcomes of Patients With DM

**FIGURE 5:**
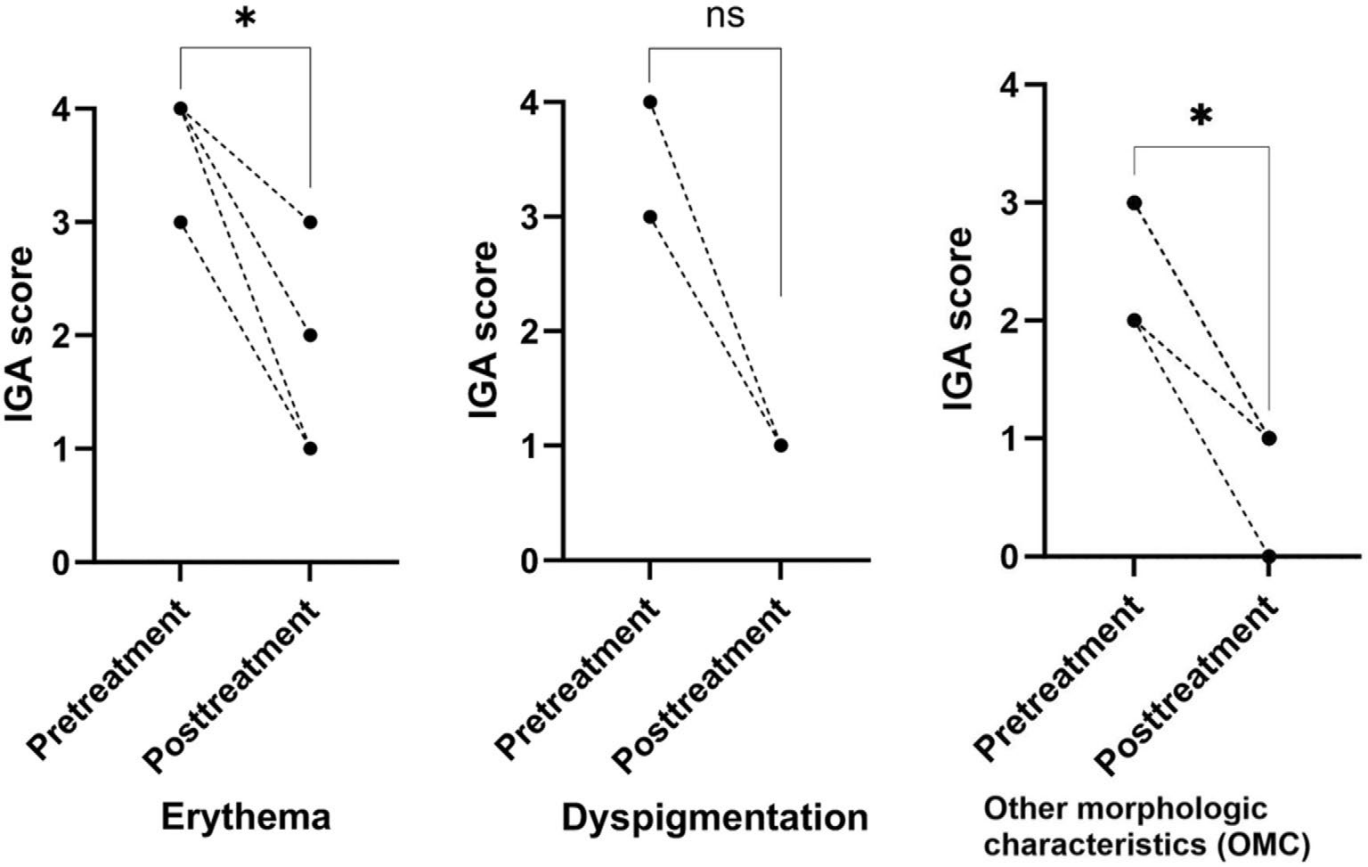
Clinical outcome of patients with dermatomyositis (DM) (*n* = 6). Changes of Investigator's Global Assessment (IGA) scores (erythema, dyspigmentation, other morphologic characteristics) before and after the treatment sessions. N‐S, not significant. *Statistically significant (*p* < 0.005) compared with baseline.

The clinical outcomes of patients with DM are presented in Figure 5. The IGA scores of erythema, dyspigmentation, and OMCs were assessed separately. Four patients with persistent facial erythema were treated with vascular lasers (PDL and LPNY laser). For these patients, we saw a significant decrease in the median (IQR) IGA score for erythema from pretreatment (4 [Q1–Q3: 3.25–4]) to posttreatment (1.5 [Q1–Q3: 1.0–2.75]) (*p* = 0.046).

Two patients were treated for postinflammatory hyperpigmentation (PIH) after their active lesions had subsided. The median (IQR) IGA score for dyspigmentation in these patients decreased from pretreatment (3.5 [Q1–Q3: 3–4]) to posttreatment (1 [Q1–Q3: 1–1]). However, this decrease was not statistically significant (*p* = 0.180).

**FIGURE 6:**
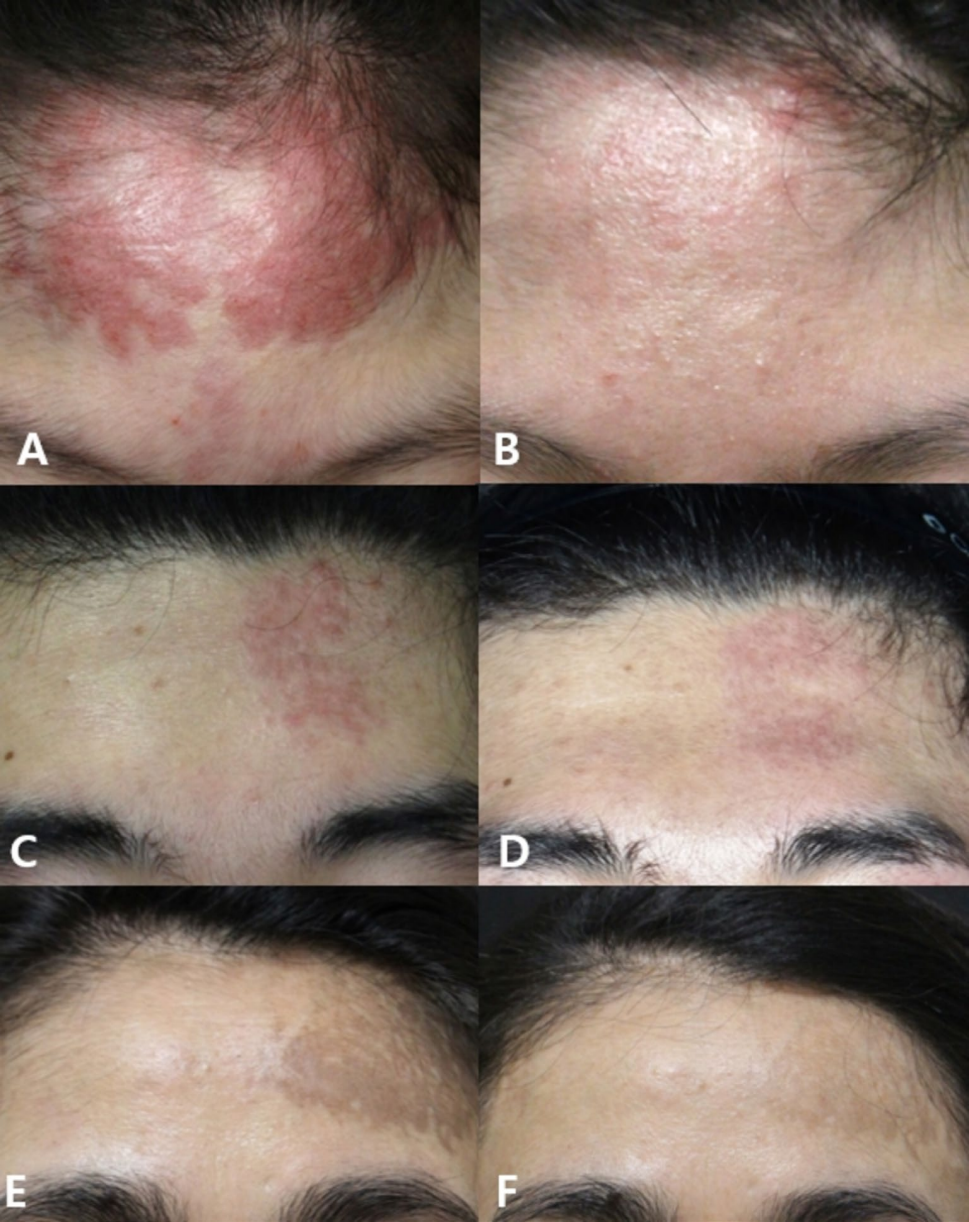
Clinical photographs of DM patients 4 (A, B), patient 3 (C, D), and patient 6 (E, F). (A, C, E) At baseline, refractory erythematous plaques or hyperpigmented patches on the forehead. (B, D, F) Notable improvement in erythema or pigmentation following EBD treatment at follow‐up (B: 12 months, D: 2 months, F: 8 months).

OMCs were evaluated in all six patients with DM. A significant decrease in the median (IQR) IGA score was observed, from 2.5 (Q1–Q3: 2–3) pretreatment to 1 (Q1–Q3: 0.75–1) posttreatment (*p* = 0.023). These results indicate that the selected laser treatments can effectively reduce erythema and improve overall skin morphology in patients with DM, although the impact on PIH may be less consistent (Figure 6).

### 3.7 Adverse Events and Cosmetic Result of Patients With DM

Overall, the treatment was well‐tolerated by patients with DM, with no reported adverse effects. The median (IQR) follow‐up period for patients with DM was 29 (Q1–Q3: 10–64) months. During the follow‐up period, none of the patients exhibited disease reactivation.

Patient‐rated cosmetic results were available for three patients. The mean patient‐reported VAS score was 5.50 ± 0.70, which is lower than that of the patients with LE. Due to incomplete patient‐reported outcome data, satisfaction results should be interpreted descriptively rather than as confirmatory evidence.

## 4 Discussion

This study included 20 LE and 6 DM patients whose cutaneous lesions persisted despite conventional therapies.

PDL was the most frequently used laser modality, employed in 21 of 26 cases (80.8%); PDL was used as an adjunctive treatment for conditions such as CLE, telangiectasia in patients with SLE, and refractory erythema often seen in patients with DM. The efficacy of PDL in treating LE has been well established, which led to an international consensus guideline recommending PDL as a fourth‐line treatment option for refractory, inactive cases of DLE [13, 14, 15]. PDL's therapeutic applications in CTDs extend beyond LE. Previous case reports and series have demonstrated the efficacy of PDL in patients with DM [6, 7, 16, 17]. One suggested mechanism behind the therapeutic effects of PDL is the selective destruction of the cutaneous microvasculature, inhibiting the migration of inflammatory cells and leading to lesion regression [18]. In addition to PDL, IPL treatment was employed in four of 20 patients with LE (20.0%) who presented with persistent facial erythema and telangiectasia during the study period. IPL has a broad wavelength spectrum, ranging from 515 to 1200 nm. Thus, IPL has wide‐therapeutic range, including pigment lesions, erythema, telangiectasia, photo‐rejuvenation, and hair removal [19, 20]. IPL treatment has been shown to manage chronic facial erythema in SLE, subacute CLE/DLE, DM, and microstomia in systemic sclerosis. Furthermore, LPNY laser was used in eight out of 26 patients (30.8%). With a wavelength of 1064 nm, LPNY laser is highly absorbed by hemoglobin and can penetrate deeper into the dermis, making it suitable for the treatment of deeper vascular lesions. Successful treatment of refractory DLE using 1064 nm LPNY laser has been reported previously [21]. Owing to its relatively low absorption by epidermal melanin, it provides deeper dermal penetration and is associated with fewer side effects in patients with darker skin types [22]. Notably, PDL was not available in our hospital until 2022, which explains why LPNY laser was predominantly used for vascular laser treatments before this period. Although we were unable to pinpoint the specific impact of LPNY laser treatment, given its combined use with other lasers, we observed a significant decrease in erythema IGA scores in both LE and DM patients, suggesting a beneficial effect of combining various vascular lasers in CTD treatment.

Dyspigmentation, as reflected in skin outcome measures of LE and DM, signifies disease damage [23]. In our study, QSNY laser was used in 20.0% (5 of 20) of patients with lupus and 33.3% (2 of 6) of patients with DM. A previous study demonstrated improvements in hyperpigmentation in a patient with DLE following treatment with a 1064 nm QSNY laser [24]; however, literature on the use of lasers to treat hyperpigmentation in patients with LE is limited. We observed a slight improvement in pigmentation in a patient treated with QSNY laser. Given that hyperpigmentation in DLE lesions is composed of both epidermal and dermal melanin, the efficacy of 532 nm QSNY laser may be limited owing to its low penetration depth [25]. However, we observed a significant decrease in dyspigmentation IGA scores when patients were treated with a combination of 532 nm QSNY laser and various other lasers. Regarding DM patients, we noticed a slight improvement in PIH in two patients after multiple treatment sessions with low‐fluence QSNY laser, although it was not statistically significant.

The application of fully ablative lasers in CTD could potentially induce complications due to skin fragility. Consequently, fractional ablative and nonablative lasers are presumed to yield fewer side effects [3]. Reports on the use of AFL in CTD have mostly focused on the treatment of morphea [3, 4, 5, 26]. In our study, AFL was used in 35.0% (7 of 20) of patients with lupus and 16.7% (1 of 6) of patients with DM, often combined with various other lasers. In every case, there was a significant improvement in OMCs. In a case where only a CO_2_ AFL was used, we observed a nearly complete clearance of DLE lesions in a patient with SLE (Figure 3C,D). Furthermore, signs of follicular activity (follicular hyperkeratosis and plugging) improved in eight patients with LE (40.0%).

When considering patient satisfaction, the overall cosmetic result using a VAS was fair, with patients with LE and DM rating their satisfaction at 6.66 ± 2.0 and 5.50 ± 0.70, respectively.

However, there are significant concerns when using lasers for the treatment of CTDs, including photosensitivity, skin fragility, and the potential exacerbation of pre‐existing diseases. CTDs, particularly LE, are often associated with photosensitivity [4, 27]. Ultraviolet A and B light can provoke or exacerbate symptoms of LE and other CTDs. However, previous studies have found that lasers within visible light wavelengths, including PDL, argon, and IPL with a 515 nm filter, are unlikely to cause photosensitivity [28, 29]. This suggests that laser treatments may be a safe, viable therapeutic option for CTDs. Throughout our study, patients with concurrent SLE did not experience disease reactivation, which further underscores this potential.

In terms of safety, we noted transient side effects in three patients, but most patients tolerated the procedures well. Even when ablative lasers were used, we did not observe any cases of skin fragility or impaired wound healing.

Despite the encouraging results of this study, several limitations need to be acknowledged. This study has several important limitations. First, the retrospective and uncontrolled design precludes causal inference. Second, the use of multiple EBD modalities across heterogeneous subtypes introduces variability that limits conclusions regarding the effectiveness of individual devices. Third, the small number of DM patients (*n* = 6) limits statistical power, and findings in this subgroup should be regarded as exploratory. Fourth, the modified IGA scale lacks external validation, reducing reproducibility and comparability. Fifth, inter‐rater reliability, although acceptable, may still introduce subjectivity. Also, patient‐reported outcomes were incomplete, and subgroup analyses were limited by small sample sizes.

Our findings should be interpreted as an expanded Korean case series demonstrating real‐world use of multiple EBDs rather than as definitive evidence of efficacy. While EBD use in LE and DM has been previously reported, our study represents one of the largest patient numbers described to date and provides longitudinal safety observations.

## 5 Conclusion

This retrospective study assessed the efficacy and safety of various laser treatments for cutaneous manifestations in patients with LE and DM. Positive cosmetic outcomes and overall tolerability were observed. The study suggests that combination laser therapy is promising for managing persistent lesions, enhancing the quality of life for CTD patients. However, given the methodological limitations, these findings need further confirmation in prospective, controlled studies.

## Funding

The authors have nothing to report.

## Ethics Statement

Informed consent was obtained from all participants. All patients provided written informed consent for the publication of their case details.

## Conflicts of Interest

The authors declare no conflicts of interest.

## Supporting information


**Table S1:** Evaluation of cutaneous manifestations in patients with LE using a modified IGA scale.
**Table S2:** Evaluation of cutaneous manifestations in patients with DM using a modified IGA scale.
**Table S3:**. List of abbreviations used in this study.
**Table S4:** Detailed parameters of energy‐based devices (EBDs) used in this study.

## Data Availability

The data that support the findings of this study are available on request from the corresponding author. The data are not publicly available due to privacy or ethical restrictions.
