# Supplementary material for: Energy‐Based Devices for the Treatment of Cutaneous Lesions in Patients With Lupus Erythematosus and Dermatomyositis
Source: J Cosmet Dermatol. 2026 Jan 12;25(1):e70642. doi: 10.1111/jocd.70642 (PMC12793946; doi:10.1111/jocd.70642)
Supplement: Supplementary file 1 — Table S1: Evaluation of cutaneous manifestations in patients with LE using a modified IGA scale. Table S2: Evaluation of cutaneous manifestations in patients with DM using a modified IGA scale. Table S3:. List of abbreviations used in this study. Table S4: Detailed parameters of energy‐based devices (EBDs) used in this study. [file JOCD-25-e70642-s001.docx]

**Supplementary Table S1. Evaluation of cutaneous manifestations in patients with LE using a modified IGA scale**

| **Patient** | **Age** | **Sex** | **Diagnosis** | **Location** | **Lesion description** | **IGA (0-4)**  **(Erythema/Dyspigmentation/OMC)** |
| --- | --- | --- | --- | --- | --- | --- |
| 1 | 31 | M | CCLE | Left cheek | DLE/LEP: Two well-demarcated erythematous indurated plaques, atrophy | 4/-/3 |
| 2 | 41 | F | CCLE | Glabella Both cheek | DLE/LEP: Four brownish depressed lesions on glabella, atrophic scar on right cheek, one brownish patch on left cheek | -/3/2 |
| 3 | 36 | F | CCLE | Forehead Both cheek Neck | Multiple erythematous patches, diffuse telangiectasia on face and neck | 3/-/2 |
| 4 | 34 | F | CCLE, SLE | Both cheek | Violaceous red patches, indurated plaque, atrophy, diffuse telangiectasia on face | 4/3/3 |
| 5 | 27 | M | CCLE | Right cheek | DLE: One erythematous indurated plaque, atrophy | 3/-/3 |
| 6 | 54 | M | CCLE | Both cheek | DLE: Two erythematous scaly plaques surrounded by brownish margin with follicular plugging | 4/3/3 |
| 7 | 23 | F | CCLE | Forehead Both cheek Chin, Nose | DLE: Multiple red to violaceous scaly papules, plaques with brownish margin | 4/3/3 |
| 8 | 37 | F | SLE telangiectasia | Left lower eyelid Nose, Right Cheek, Perioral area | Multiple telangiectatic patches | 3/-/1 |
| 9 | 22 | F | CCLE, SLE | Both postauricular | DLE: Three distinct erythematous scaly plaques with peripheral pigmentation. | 4/4/3 |
| 10 | 27 | F | CCLE, SLE | Forehead, Glabella Both cheek, Chin | Multiple erythematous edematous plaques, with some atrophic scars | 4/-/2 |
| 11 | 40 | M | CCLE, SLE | Nose Both cheek | DLE: Multiple erythematous plaques, with some atrophic scars | 3/4/3 |
| 12 | 32 | M | CCLE, SLE | Forehead Nose Both cheek | DLE: Erythematous indurated plaques, with prominent secondary changes (erosion, crusting, scarring) | 4/-/4 |
| 13 | 30 | F | SLE telangiectasia | Both cheek | Diffuse telangiectasia on face | 3/-/0  -  0 |
| 14 | 29 | F | CCLE, SLE | Forehead,  Both postauricular | Erythematous patches with telangiectasia along the forehead hairline and postauricle | 3/-/2 |
| 15 | 40 | M | CCLE | Forehead Nasal dorsum Both cheek | DLE: Multiple erythematous edematous plaques | 3/-/3 |
| 16 | 36 | F | CCLE, SLE | Nasal dorsum | Solitary erythematous indurated plaque | 3/-/2  2 |
| 17 | 45 | F | SLE telangiectasia | Both cheek | Diffuse telangiectasia on face | 2/-/0  -  0 |
| 18 | 37 | F | CCLE, SLE | Forehead Nose, Philtrum | DLE: Three darkly pigmented, scaly papules | -/4/3 |
| 19 | 34 | F | SLE telangiectasia | Right lower eyelid Philtrum | Multiple telangiectatic patches | 3/-/0 |
| 20 | 32 | F | CCLE, SLE | Right cheek | Solitary erythematous indurated plaque | 3/-/2 |
|  | | | | | | |

**Supplementary Table S2. Evaluation of cutaneous manifestations in patients with DM using a modified IGA scale**

| **Patient** | **Age** | **Sex** | **Diagnosis** | **Location** | **Lesion description** | **IGA (0-4)**  **(Erythema/Dyspigmentation/OMC)** |
| --- | --- | --- | --- | --- | --- | --- |
| 1 | 49 | F | Adult DM | Forehead Eyelid  Cheek | Post-inflammatory hyperpigmentation after the erythema subsided | -/3/3 |
| 2 | 24 | M | Juvenile DM | Right cheek | Solitary erythematous indurated plaque with hypopigmentation, atrophic scar | 4/-/3 |
| 3 | 17 | M | Juvenile DM | Forehead Nasal dorsum | Erythematous indurated plaques | 3/-/2 |
| 4 | 11 | F | Juvenile DM | Forehead Both cheek | Erythematous indurated plaque on forehead, erythematous patch along the malar area | 4/-/2 |
| 5 | 42 | F | Adult DM | Forehead Both temple Both cheek | Multiple erythematous edematous plaques | 4/-/3 |
| 6 | 48 | F | Adult DM | Forehead  Eyelid | Post-inflammatory hyperpigmentation after the erythema subsided | -/4/2 |
|  | | | | | | |

**Supplementary Table S3. List of Abbreviations used in this study**

| **Abbreviations** | **Full term** |
| --- | --- |
| **AZA** | Azathioprine |
| **BEL** | Belimumab |
| **CLC** | Colchicine |
| **CSA / CsA** | Cyclosporine A |
| **DEF** | Deflazacort |
| **HCQ** | Hydroxychloroquine |
| **MMF** | Mycophenolate Mofetil |
| **MTX** | Methotrexate |
| **PDS** | Prednisolone |
| **TAC** | Tacrolimus |
| **TA** | Triamcinolone |
| **AFL** | Ablative Fractional Laser |
| **Er:YAG** | Erbium-Doped Yttrium Aluminum Garnet |
| **H-lase** | Hyaluronidase |
| **IPL** | Intense Pulsed Light |
| **LPNY** | Long-Pulsed Nd:YAG |
| **Nd:YAG** | Neodymium-Doped Yttrium Aluminum Garnet |
| **PDL** | Pulsed Dye Laser |
| **QSNY** | Q-Switched Nd:YAG |
| **EBD** | Energy-Based Device |
| **HA** | Hyaluronic Acid |
| **HQ** | Hydroquinone |
| **IGA** | Investigator Global Assessment |
| **ILI** | Intralesional injection |
| **OMC** | Other Morphological Characteristics |
| **PDRN** | Polydeoxyribonucleotide |
| **TCI** | Topical Calcineurin Inhibitor |
| **TCS** | Topical Corticosteroid |
| **VAS** | Visual Analog Scale |
|  |  |
|  | |

**Supplementary Table S4. Detailed Parameters of Energy-Based Devices (EBDs) Used in This Study**

| **Device Type** | **Laser/Device** | **Wavelength(s)** | | **Spot size** | **Pulse Duration** | **Fluence** | **Other Parameters** | **Manufacturer (Model)** |
| --- | --- | --- | --- | --- | --- | --- | --- | --- |
| **Vascular Laser** | PDL | 595 nm | | 7 mm | 3.0–10.0 ms | 5.0–12.0 J/cm² | DCD 30/20 | Candela (Vbeam Perfecta, USA) |
|  | LPNY | 1,064 nm | | 1.5 mm / 3.0 mm | 12.0–25.0 ms | 117.0–365.9 J/cm² | — | Ellipse Flex System (Ellipse A/S, Denmark) |
| **Broadband Light** | IPL (VL applicator) | 555–950 nm | | — | 8.0–10.0 ms | 6.0–13.3 J/cm² | — | Ellipse Flex System (Ellipse A/S, Denmark) |
| **Pigment Laser** | QSNY | 532 / 1,064 nm | | 3–10 mm | — | 0.8–2.0 J/cm² | — | Lutronic (Spectra XT plus, Korea) |
| **Ablative Fractional** | CO₂ AFL | — | | 120 μm (tip size) | — | — | 30–45 mJ, 30 W, 100–200 spots/cm² | Lutronic (eCO₂, Korea) |
|  | Er:YAG AFL | 2,940 nm (Er:YAG) | | 10 mm | — | 12.0–14.0 J/cm² | — | Lutronic (Action Spectra, Korea) |
|  | | |  | | | | | |
